# Predictive factors for running economy in female recreational runners

**DOI:** 10.1016/j.clinsp.2026.101158

**Published:** 2026-09-19

**Authors:** Fabio de Paula Domingos, Vinícius Ribeiro dos Anjos Souza, Lavínia Vivan, Claudio Andre Barbosa de Lira, Rodrigo Luiz Vancini, Katja Weiss, Beat Knechtle, Marilia Santos Andrade

**Affiliations:** a Post-graduate Program in Translation Medicine, Universidade Federal de São Paulo, São Paulo, SP, Brazil; b Human and Exercise Physiology Division, Faculty of Physical Education, Universidade Federal de Goiás (UFG), Goiania, GO, Brazil; c Center for Physical Education and Sports, Universidade Federal do Espírito Santo (Ufes), Vitória, ES, Brazil; d Institute of Primary Care, University of Zurich, Zurich, Switzerland; e Department of Physiology, Universidade Federal de São Paulo (UNIFESP), São Paulo, SP, Brazil

**Keywords:** Aerobic capacity, Body composition, Energy cost, Running economy, V̇O_2_max

## Abstract

**Purpose:**

To assess the predictive value of age, sport experience, lower-limb flexibility, lean mass, Fat percentage (%FAT), Maximal Oxygen consumption (V̇O_2_max), knee extensor muscle Peak Torque (PT), and Maximal Aerobic Speed (MAS) on Running Economy (RE) in female long-distance recreational runners.

**Methods:**

A total of 61 female recreational runners aged 20–60 yr participated in this study. RE was evaluated based on the Energy Cost of running (EC). Additional parameters measured included V̇O_2_max, flexibility, lean mass, %FAT, MAS, sport experience, age, and PT.

**Results:**

EC was significantly associated with %FAT (*r* = 0.629, p < 0.001), flexibility (*r* = -0.388, p = 0.002), lean mass (*r* = -0.330, p = 0.009), and MAS (*r* = -0.516, p < 0.001). No significant correlations were observed between RE and age (*r* = 0.017, p = 0.896), sport experience (*r* = -0.119, p = 0.360), V̇O_2_max (*r* = -0.200, p = 0.121), or PT (*r* = -0.026, p = 0.841). Regression analysis revealed that %FAT (β = 0.017, *t* = 4.168, p < 0.001) and MAS (β = -0.039, *t* = -1.987, p = 0.052) accounted for 43.5% of the variation in EC.

**Conclusion:**

These findings indicate that %FAT and MAS were the key predictors of RE in female recreational runners, with higher %FAT associated with poorer RE and greater MAS correlating with improved RE. However, MAS showed a borderline association with EC (p = 0.052), suggesting a potential but cautious interpretation as a predictor. These insights underscore the importance of training strategies that optimize body composition and aerobic performance to enhance RE in female recreational runners.

## Highlights

• RE correlated with %FAT (+), flexibility (-), lean mass (-), and MAS (-).
• No correlation between RE and age, sport experience, VO_2_max, or PT.
• %FAT and MAS explained 43.5% of EC variation in female runners.

## Introduction

Running performance progressively deteriorates with age, particularly after 30–34 yr^1^^,^^2^ This decline is often attributed to reductions in Maximal Oxygen consumption (V̇O_2_max), but V̇O_2_max alone does not fully explain performance outcomes^3^ Running Economy (RE) is another crucial determinant of endurance performance, yet it has received relatively less attention in research^4^, ^5^, ^6^ Various factors influence RE, including age, flexibility, fitness level, muscle strength, body composition, mechanics, and cardiorespiratory function^7^^,^^8^ While RE does decline with age, this decrease is generally less severe than the decline in V̇O_2_max^9^

Despite a significant increase in female participation in endurance sports, in recent years,^7^ scientific research remains predominantly centered on the male population^10^ Consequently, findings primarily based on male athletes are often applied to female athletes without adequate scientific validation^11^^,^^12^

Given these considerations, the present study aimed to examine the potential associations between age, body composition, flexibility, strength, and aerobic fitness level with RE based on Oxygen Cost (OC) and Energy Cost (EC) in female recreational runners. Additionally, we sought to develop a predictive equation for Oxygen Cost (OC) and Energy Cost (EC) based on these independent variables.

We hypothesized that body composition (lower %FAT and higher lean mass) and Maximal Aerobic Speed (MAS) would be the primary predictors of RE, whereas age and concentric muscle strength would not show significant associations.

## Materials and methods

### Ethical considerations

The study protocol was reviewed and approved by the Human Research Ethics Committee of the Federal University of São Paulo (UNIFESP) under approval number 0765/2023, dated November 19, 2023. The study adhered to the ethical principles outlined in the Declaration of Helsinki. Prior to participation, all athletes received a comprehensive explanation of the study’s objectives, experimental protocols, potential risks and benefits, and assurances regarding privacy, confidentiality, and anonymity. Each participant provided written informed consent before enrollment.

### Participants

Participants were recruited through sports consultants, running coaches, athletic clubs, and social media platforms. The study sample comprised female recreational runners aged 20 to 60 yr. A total of 61 volunteers participated, with a mean age of 40.9 ± 11.7 yr, a mean body mass of 59.94±7.91 kg, and an average height of 1.631±0.062 m. The minimal sample size calculated a priori was 59 participants, as described below.

Inclusion criteria were being a woman who has been running for more than 2-years, with a minimum frequency of 3-times a week.

Exclusion criteria included medical contraindications to maximal effort; a history of smoking; myopathies; systemic inflammatory or lung parenchymal diseases; cardiomyopathies; peripheral vascular diseases; and orthopedic limitations that could impede maximal effort during the assessments. The descriptive characteristics of the participants are presented in Table 1. Participants reported running training an average of 4.1 ± 1.1 days per week, with a mean session duration of 74.1 ± 26.8 min. Average weekly running volume was 42.2 ± 21.8 km. Additionally, the best self-reported personal time for the 10 km distance was 56.5 ± 13.2 min. Only one participant did not engage in strength training in addition to her running training. Among those who performed strength training, the average frequency was 2.7 ± 1.6 sessions per week, with a mean duration of 59.3 ± 22.2 minutes per session.

**Table 1:** Characteristics of the participants.

	Mean ± SD
**Age (yr)**	40.9 ± 11.7
**Body mass (kg)**	59.94 ± 7.91
**Height (m)**	1.631 ± 0.062
**Sport experience (yr)**	9.6 ± 6.7
**Lean mass (kg)**	39.564 ± 3.593
**Flexibility (cm)**	28.46 ± 9.24
**%FAT (%)**	28.852 ± 8.114
**V̇O_2_max (mL·kg^-^¹·min^-^¹)**	45.872 ± 6.971
**MAS (km·h^-^¹)**	14.2 ± 1.7
**PT (Nm·kg^-^¹)**	123.78 ± 24.49

%FAT, Fat percentage; MAS, Maximal Aerobic Speed; PT, Peak of Torque; SD, Standard Deviation; V̇O_2_max, Maximal oxygen consumption.

### Study design

The assessments were conducted during a single visit to the Exercise Physiology Laboratory at the Federal University of São Paulo (UNIFESP). This is a cross-sectional study conducted in accordance with the STROBE guidelines. Before undergoing testing, participants completed a questionnaire, as described below. Following this, flexibility, isokinetic knee strength, anthropometric data, RE, V̇O_2_max, and Maximal Aerobic Speed (MAS) were measured in this order. After the isokinetic strength assessment, participants underwent Dual-energy X-Ray Absorptiometry (DXA) and remained in a supine position for approximately 15–20 min during the scan. The running economy assessment was then performed. Following a standardized 10-min seated recovery period, participants completed the cardiopulmonary exercise test. The RE test was performed prior to the cardiopulmonary exercise test for V̇O_2_max and MAS determination to avoid any potential influence of fatigue from the maximal test on RE measurements. Given that the RE test was conducted at intensities below the first ventilatory threshold, it did not induce fatigue capable of affecting the subsequent maximal test^13^ A schematic flowchart of the experimental design is presented in Fig. 1.

**Fig. 1:**
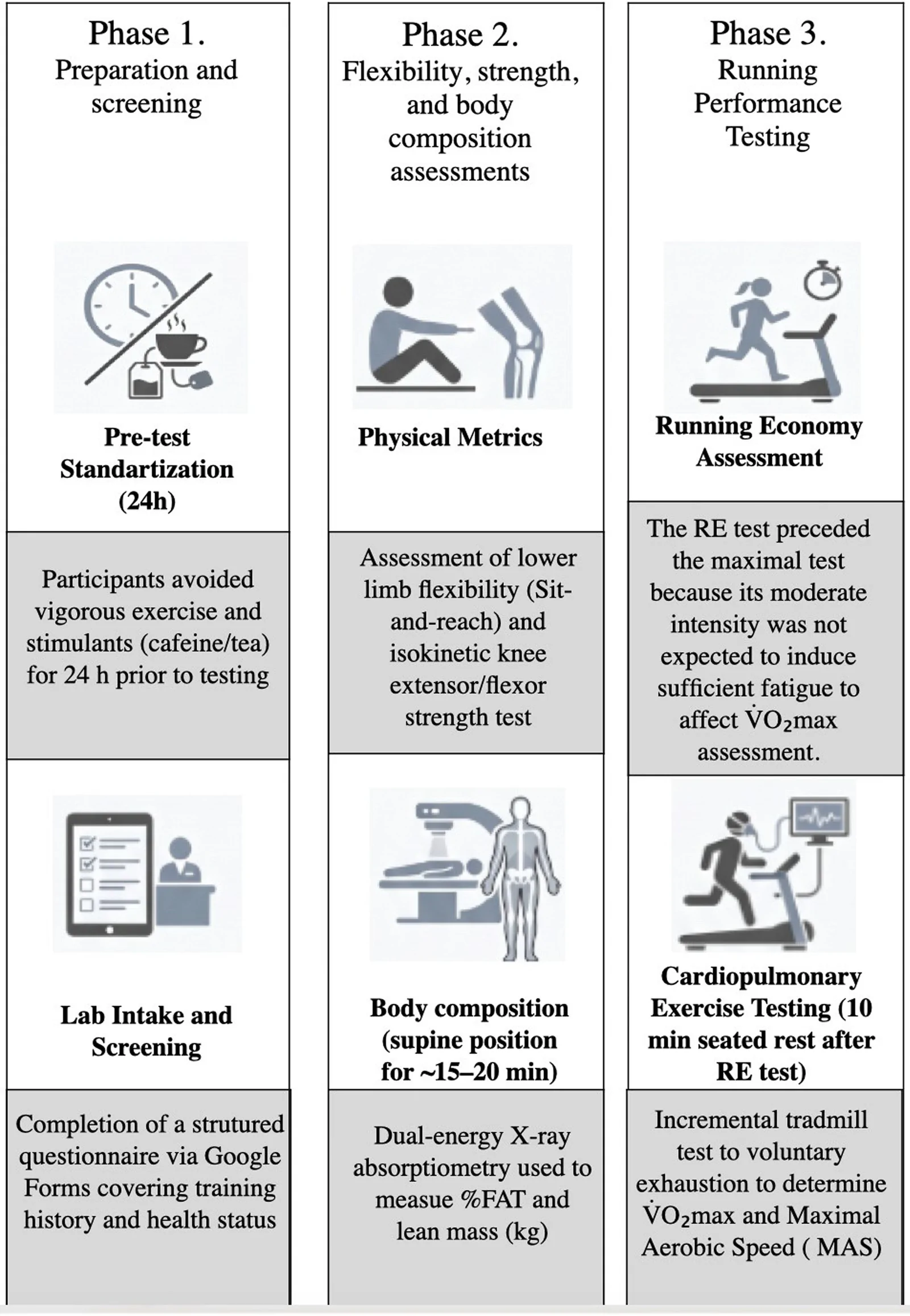
Schematic flowchart of the experimental design. Generative artificial intelligence (ChatGPT, OpenAI) was used to assist in the design and layout of the figure. The final figure was reviewed and verified by the authors.

Participants were instructed to refrain from strenuous exercise and avoid consuming supplements or stimulant-containing foods and beverages (e.g., caffeine and tea) for at least 24 h before testing. All participants wore lightweight, comfortable clothing, and were advised to wear the same running shoes they typically used for training sessions.

### Experimental procedures

#### Questionnaire

A structured questionnaire was administered via Google Forms (Google LLC, Mountain View, USA), consisting of nine open-ended questions: How long have you been running (yr)? How many times per week do you run? What is your average weekly running volume (km)? Have you ever sustained a running-related injury? If yes, specify. Are you currently unable to run due to an injury? Do you have any of the following chronic illnesses: myopathies, systemic inflammatory diseases, lung parenchymal diseases, cardiomyopathies, peripheral vascular diseases, or orthopedic limitations? Are you a smoker? Do you experience chest pain and/or dizziness during physical activity? Has a doctor ever advised you against engaging in physical activity?

#### Body composition assessment (%FAT and lean mass)

Participant’s body height and total body mass were measured using a calibrated wall stadiometer and a balance (Filizola®, São Paulo, Brazil), respectively. Measurements were recorded to the nearest 0.1 cm and 0.1 kg. Body composition (lean mass and %FAT) was measured using dual-energy X-Ray absorptiometry (software version 12.3; Lunar DPX, Wisconsin, USA). This method is highly reliable for assessing body composition with minimal radiation exposure (< 10 microSieverts)^14^

#### Assessment of flexibility

Flexibility was assessed using the sit-and-reach test, performed on a Wells bench according to the protocol established by the authors^15^

#### Isokinetic muscular strength assessment

The thigh muscle strength of the participants was assessed using an isokinetic strength test, performed using a Biodex System 3 isokinetic dynamometer (Biodex Medical System, New York, USA) to measure the concentric strength of the dominant lower limb, identified as the participant’s preferred kicking leg^16^

Knee extensor strength was assessed over a 90° range of motion, with gravity correction applied at full knee extension. To familiarize participants with the test, they performed three submaximal practice trials at 50%–75% of their maximum effort. Stretching exercises were avoided, as they can influence the strength results^17^ Knee assessment was performed following the manufacturer’s instructions, with participants seated on the dynamometer chair, trunk upright, and hip flexed at 85°. The knee axis was aligned at 90° using the lateral femoral epicondyle as a landmark and matched with the dynamometer shaft. The knee attachment was positioned near the malleolus and secured with straps, while the trunk and hip were stabilized with Velcro straps prior to testing. The actual test consisted of five maximal effort repetitions in concentric mode at an angular velocity of 60°/s.

To ensure data reliability, results with a coefficient of variation exceeding 10% were excluded. Standardized verbal encouragement was provided to maximize participant effort. The primary outcome measures were Peak Torque (PT) in Nm and PT normalized to body mass (Nm/kg^–1^) for both knee flexor and extensor muscles.

#### Running economy assessment

RE was assessed using a treadmill (Imbrasport, ATL, Porto Alegre, Brazil) while physiological responses to exercise were continuously monitored with a metabolic system (Quark PFT, Cosmed®, Rome, Italy).

Expired gas samples and an expiratory flow sensor provided real-time measurements of Oxygen consumption (V̇O_2_), Carbon dioxide production (V̇CO_2_), and Respiratory Quotient (RQ). The system was calibrated prior to testing according to the manufacturer’s specifications.

The assessment protocol included a one-minute baseline rest period to stabilize heart rate and pulmonary ventilation. Participants then ran for a total of 8-min, with two distinct phases: an initial 4-min segment at a steady speed of 8 km/h^–1^, followed by another 4-min phase at 9 km/h. The fixed running speeds of 8 and 9 km/h^–1^ were selected to provide standardized submaximal workloads suitable for recreational female runners. These speeds were chosen to allow the assessment of RE at low-to-moderate exercise intensities. To confirm the submaximal nature of the protocol, the speeds used during the RE test were compared with the first ventilatory threshold subsequently determined during the CPET and were confirmed to be below this threshold in all participants. RE was quantified using the Oxygen Cost (OC) and Energy Cost (EC) metric. EC was determined from the mean V̇O_2_ during the final minute of each running segment. RQ values were incorporated to estimate the metabolic substrate utilization. Using non-protein RQ equations, the relative contributions of fat and carbohydrate oxidation were calculated, with energy equivalents of 9.75 kcal/g^–1^ for fats and 4.07 kcal/g^–1^ for carbohydrates applied^18^ Energy cost (EC; kcal/kg^–1^/km^–1^) has been recommended in the scientific literature as the most accurate method for assessing RE, as it incorporates the Respiratory Exchange Ratio (RER) to estimate the proportion of substrates (carbohydrates and fats) utilized during exercise. By accounting for these variations in substrate oxidation, EC provides a more precise quantification of the actual metabolic expenditure compared to the traditional Oxygen Cost (OC; mL/kg^–1^/km^–1^), which is less sensitive to changes in substrate utilization and may therefore underestimate or overestimate the metabolic demand under different physiological conditions^19^, ^20^, ^21^, ^22^ Therefore, for the purposes of data analysis and discussion, RE will be interpreted and discussed in terms of energy cost. The oxygen cost data are presented here solely to allow readers to make comparisons with other studies that have reported RE based on oxygen cost rather than energy cost.

#### Cardiopulmonary exercise testing

A Cardiopulmonary Exercise Test (CEPT) was performed on the same treadmill and metabolic system to evaluate maximal aerobic capacity. The protocol began with a 1-min resting period, followed by a standardized 3-min warm-up at a constant speed of 8 km/h^–1^, with speed increasing by 1 km/h per minute until voluntary exhaustion^23^ The treadmill incline was maintained at 1% throughout the test^23^

The Borg scale was used to assess participants’ perceived exertion at the end of each stage^24^ V̇O_2_max was determined based on the occurrence of a plateau in V̇O_2_^25^ If no plateau was observed, V̇O_2_max was defined according to the following secondary criteria: a respiratory exchange ratio ≥ 1.10, achievement of age-predicted maximum heart rate, and a Borg scale rating of 20. The first Ventilatory Threshold (VT) was determined according to established ventilatory criteria widely used in the literature,^25^ based on the following criteria: an inflection in the ventilation curve, an increase in the Ventilatory Equivalent for Oxygen (V̇E/V̇O₂) without a concomitant increase in the ventilatory equivalent for carbon dioxide (V̇E/V̇CO₂), and an increase in the partial pressure of exhaled oxygen (PetO₂). VT was independently determined by two experienced investigators. In cases of disagreement, a third investigator was consulted to establish the final VT.

Maximum Aerobic Speed (MAS) was identified as the highest running speed attained during the final stage of the test^26^

### Statistical analysis

Descriptive data are presented as means and standard deviations. The sample size for the predictive model was determined a priori. An effect size of 0.30, representing a moderate effect,^27^ was used, with a significance level of 0.05 and a statistical power of 0.80. The results indicated that a minimum sample size of 59 participants was required.

To assess relationships among variables, Pearson’s correlation analysis was performed, and it was classified as “very weak” when *r* = 0.0 to 0.19, “weak” when *r* = 0.20 to 0.39, moderate” when *r* = 0.40 to 0.59, “strong” when *r* = 0.60 to 0.79, and “very strong” when *r* = 0.80 to 1.0^28^ Significant correlations were included in a stepwise multiple linear regression model.

For the regression models, the coefficient of determination (*r*^2^), was reported to evaluate the model’s predictive strength. Additionally, the Durbin-Watson test was used to assess autocorrelation, while Variance Inflation Factor (VIF) and tolerance values were examined to detect multicollinearity. The normality of residuals was also assessed. To assess the stability of the final regression model, a sensitivity analysis was performed using bootstrap resampling with 10,000 iterations and 95% Confidence Intervals. All statistical analyses were performed using Jamovi software (version 2.4.8.0), with a significance threshold of p < 0.05.

## Results

The correlation analysis results between OC and EC and independent variables are presented in Table 2. Significant correlations were observed between EC and the following variables: %FAT (*r* = 0.629, p < 0.001), flexibility (*r* = −0.388, p = 0.002), lean mass (*r* = −0.330, p = 0.009), and MAS (*r* = −0.516, p ≤ 0.001). However, EC did not show statistically significant correlations with age (*r* = 0.017, p = 0.896), sports experience (*r* = −0.119, p = 0.360), V̇O_2_max (*r* = −0.200, p = 0.121), and PT (*r* = −0.026, p = 0.841).

**Table 2:** Association level between energy cost and the other independent variables.

	Age (yr)	Sport experience (yr)	VO_2_max (mL/kg^–1^/min^-^¹)	Lean mass (kg)	Flexibility (cm)	%FAT (%)	MAS (km/h^–1^)	PT (Nm/kg^–1^)	EC (kcal/kg^–1^/km^–1^)	OC (mL/kg^–1^/km^–1^)
Age (yr)	‒	0.607	−0.487	0.063	−0.034	0.023	−0.440	−0.353	0.017	−0.007
Sports experience (yr)		‒	−0.169	−0.001	0.018	−0.086	0.156	−0.323	−0.119	−0.200
V̇O_2_max (mL/kg^–1^/min^–1^)			‒	0.051	0.248	−0.551	0.690	0.351	−0.200	0.382^a^
Lean mass (kg)				‒	0.16	−0.258	0.115	0.380	−0.330^a^	−0.152
Flexibility (cm)					‒	−0.390	0.333	0.102	−0.388^a^	−0.103
%FAT (%)						‒	−0.561	0.034	0.630^a^	−0.261^a^
MAS (km/h^–1^)							‒	0.338	−0.516^a^	−0.006
PT (Nm/kg^–1^)								‒	−0.026	−0.054
EC (kcal/kg^–1^/km^–1^)									‒	0.564^a^
OC (mL/kg^–1^/km^–1^)										‒

%FAT, fat percentage; EC, Energy Cost of running; MAS, Maximal Aerobic Speed; PT, Peak of Torque; V̇O_2_max, Maximal oxygen consumption; OC, Oxygen Cost of running.

a p < 0.05.

Regarding OC significant correlations were observed between OC and V̇O_2_max (*r* = 0.382, p = 0.002) and %FAT (*r* = −0.261, p = 0.042).

To identify the best anthropometric and physiological predictors of EC and OC among female recreational runners, stepwise multiple linear regression was performed. The final regression models are summarized in Table 3.

**Table 3:** Multiple regression for estimating participant EC and OC.

	*r*^2^	*Z*	*df*	p-value	SEE	Tolerance	VIF	Durbin-Watson
	*EC* = 1.909 + 0.017 × (%FAT) − 0.039 × (MAS)							
**EC**	0.435	23.86	2, 60	<0.001	0.21	0.685	1.459	2.045
	*OC* = 166.93 + 1.394 × (V̇O_2_max)							
**OC**	0.146	10.06	2, 60	0.002	23.74	1.0	1.0	1.899

%FAT, Fat percentage in percentage; EC, Energy Cost of running in kcal.kg^-^¹.km^-^¹; OC, Oxygen Cost of running in mL/kg^-^¹/km^-^¹; MAS, Maximal Aerobic Speed in km.h^-^¹; PT, Peak of Torque in Nm; V̇O_2_max, Maximal Oxygen uptake in mL.kg^–1^.min^–1^; %FAT, Fat mass in percentage points.

The regression analysis identified %FAT (β = 0.017; standardized β = 0.497, *t* = 4.168, p < 0.001) and MAS (β = −0.039; standardized β = −0.237, *t* = −1.987, p = 0.052) as the most relevant predictors of EC among the variables examined in the final regression model. These two variables accounted for 43.5% of the variance in EC, highlighting the significant role of body composition and maximal aerobic speed in running efficiency among female recreational runners. Sensitivity analysis using 10,000 bootstrap resamples supported the stability of the model. The 95% bootstrap confidence interval for the MAS regression coefficient (β = −0.0398) ranged from −0.0801 to −0.0035, and the coefficient remained negative in 98.4% of the bootstrap samples.

The regression analysis identified V̇O_2_max as the only significant predictor of OC (β = 1.394; standardized β = 0.382; *t* = 3.172; p = 0.002), explaining 14.6% of the variance in OC.

## Discussion

This study aimed to determine whether age, body composition, flexibility, strength, and aerobic fitness level are associated with RE in female recreational runners. Additionally, we aimed to develop predictive models of RE based on Oxygen Cost (OC) and Energy Cost (EC) in this population. The initial hypothesis of the study was confirmed for EC but not for OC, as body fat percentage and MAS were the two variables that composed the EC prediction equation and only V̇O_2_max composed the OC prediction equation. Furthermore, age, flexibility and strength did not show a correlation with RE.

The findings indicate a negative association between lean mass and EC, meaning that higher lean mass improves RE. Conversely, %FAT showed a positive correlation with EC. Notably, the correlation between %FAT and EC were stronger than that between lean mass and EC. These results align with previous research by Saunders et al., 2004,^6^ which identified body fat and lean mass as key anthropometric factors influencing RE. Similarly, Cureton and Sparling analyzed metabolic responses during running in male and female distance runners. Their study demonstrated that women require more oxygen per unit of fat-free mass to sustain a given submaximal running speed, indicating poorer RE, which was attributed to their higher fat mass^29^ Our results align with these findings, reinforcing the importance of lower fat mass percentages in reducing EC over lean mass percentages.

The present results indicate a significant association between flexibility and EC, suggesting that female recreational runners with better lower-limb flexibility exhibit lower EC during running. Previous studies have shown that trunk and lower-limb flexibility influence RE among male athletes.^8^^,^^30^, ^31^, ^32^ Because most of the studies reviewed included only male participants, our study provides new evidence supporting the role of flexibility in improving EC among female recreational runners.

Unlike flexibility, lower-limb muscle strength did not correlate significantly with EC. These findings align with those of Li et al. 2021, who found no significant association between RE and leg press concentric peak force, 1-RM squat strength, or countermovement jump performance in male runners (5000 m, 10,000 m, and marathon). However, they reported a relationship between eccentric peak torque and RE^33^ This discrepancy suggests that strength training improves RE by enhancing eccentric muscle contractions rather than concentric strength, which was the focus of our assessment. Another possible explanation for the lack of association between peak torque and RE is that running involves movements performed at high angular velocities, with acceleration and deceleration phases, whereas peak torque assessment was conducted at a constant angular velocity of 60°/s. This discrepancy between the contraction mode used during testing and the muscular action involved in running may also have contributed to the absence of an observed association. Finally, another possible explanation for the lack of association between concentric strength and RE is the high prevalence of strength training in our sample, as 60 of the 61 participants reported engaging in strength training. This unusually high prevalence among recreational runners may have reduced the variability in strength-related characteristics within the sample and, consequently, may have influenced the observed association between concentric strength and RE.

The results showed that V̇O_2_max did not correlate significantly with EC (*r* = −0.2005, p = 0.121), whereas MAS demonstrated a strong negative correlation with EC (*r* = −0.516, p < 0.001). Previous research has established that MAS is influenced by both V̇O_2_max and RE^34^ Therefore, it is expected that MAS would be more strongly associated with EC than V̇O_2_max, making it a more effective predictor of running efficiency.

A noteworthy finding of this study was the absence of a significant association between EC and age or running experience in this cross-sectional sample of women aged 20 to 60 years. This result contrasts with some previous studies conducted in male runners[9] but may reflect sex-specific differences or methodological aspects. Further investigations are warranted to clarify whether aging influences RE in women. The final multiple regression model identified %FAT and MAS as the strongest predictors of EC, accounting for approximately 43% of its variance. Although MAS exhibited an inverse association with Energy Cost (EC) in the regression model, this relationship did not reach conventional statistical significance (p = 0.052). Therefore, the magnitude and practical relevance of MAS as an independent predictor of EC should be interpreted with caution. Moreover, the findings suggest that %FAT is a stronger predictor of EC than lean mass, emphasizing the importance of fat mass reduction for improved RE and MAS is a better predictor of EC than V̇O_2_max, reinforcing its relevance as a key performance indicator.

### Study limitations and strengths

As a cross-sectional study, our research does not allow conclusions regarding training-induced adaptations over time. Another limitation of the study is that muscle strength was assessed in isokinetic mode, at a constant angular velocity of 60°/s, which differs considerably from the type of muscular action involved in running. This discrepancy may have influenced the results observed. Another limitation of this study is that flexibility was assessed using the sit-and-reach test, which mainly reflects the flexibility of the hamstring and lower back muscles. However, running involves the coordinated action of multiple muscle groups, including the hip flexors, extensors, and plantar flexors, which were not evaluated by this test. Therefore, it is possible that flexibility assessments involving additional or more specific muscle groups could show different or stronger associations with RE. Finally, menstrual cycle phase was not controlled in the female recreational runners, which could potentially have influenced the DXA-derived body composition measurements. However, available evidence suggests that any potential influence of menstrual cycle–related hormonal fluctuations on DXA-derived body composition measurements is generally small and unlikely to be clinically meaningful^35^^,^^36^ Additionally, menopausal status and hormonal contraceptive use were not assessed in the present study. Given the potential influence of hormonal status on physiological responses, the lack of this information may represent a source of uncontrolled variability and should be considered when interpreting the findings. Although participants were instructed to consume a light meal 2–4 h before testing and avoid extreme carbohydrate manipulations (low carb or carb loading), pre-test nutritional intake was not strictly controlled and may have influenced RER measurements. However, a major strength of this study is its focus on female recreational runners, a population that remains substantially underrepresented in sports science research despite the increasing participation of women in endurance running.

### Practical applications

The findings of this study highlight the significant influence of %FAT and MAS on RE in female recreational runners. Reinforcing the importance of both to enhance RE by reducing EC.

## Conclusion

Specifically, %FAT and MAS predict 43% of EC variation, highlighting their importance in RE; fat mass reduction is more influential than lean mass gains for improving RE; and MAS is a better predictor of RE than V̇O_2_max, reinforcing its value as a training focus. However, MAS showed a borderline association with EC (p = 0.052), suggesting a potential but cautious interpretation as a predictor. These findings underscore the necessity of targeted training strategies aimed at optimizing body composition and aerobic capacity to enhance RE in female recreational runners.

## Author’s contribution

Fabio P. Domingos: Methodology; Visualization; Writing-original draft preparation.

Vinícius R.A. Souza: Data curation.

Lavínia Vivan: Data curation.

Claudio A.B. de Lira: Visualization; Investigation.

Rodrigo L. Vancini: Validation.

Katja Weiss: Writing-reviewing and editing.

Beat Knechtle: Writing-reviewing and editing.

Marilia S. Andrade: Conceptualization; Writing-reviewing and editing.

## Ethical approval statement

The study protocol was reviewed and approved by the Human Research Ethics Committee of the Federal University of São Paulo (UNIFESP) under approval number 0765/2023. The study adhered to the ethical principles outlined in the Declaration of Helsinki. All participants provided written informed consent before enrollment.

## Data availability

We will make available the data upon request to Marilia S Andrade (marilia1707@gmail.com).

## Declaration of competing interest

The authors declare no conflicts of interest.
